# Temporal expectancy induced by the mere possession of a placebo analgesic affects placebo analgesia: preliminary findings from a randomized controlled trial

**DOI:** 10.1038/s41598-022-05537-9

**Published:** 2022-01-26

**Authors:** Victoria Wai-lan Yeung

**Affiliations:** Department of Applied Psychology, and Wofoo Joseph Lee Consulting and Counselling Psychology Research Center, Lingnan University, 8 Castle Peak Road, Tuen Mun, New Territories Hong Kong

**Keywords:** Psychology, Reward

## Abstract

Research on placebo analgesia usually shows that people experienced a reduction in pain after *using* a placebo analgesic. An emerging line of research argues that, under some circumstances, *merely possessing* (but not using) a placebo analgesic could induce placebo analgesia. The current study investigates how temporary expectation of pain reduction associated with different forms of possessing a placebo analgesic affects pain outcomes. Healthy participants (*n* = 90) were presented with a vial of olive oil (placebo), described as a blended essential oil that blocks pain sensations upon nasal inhalation, and were asked to anticipate the benefits of such analgesic oil to the self (such as anticipating the analgesic oil to reduce their pain). Participants were randomized into one of three different possession conditions: *physical-possession condition* (participants possessed a tangible placebo analgesic oil, inducing an expectation to acquire analgesic benefit early upon the experience of pain), *psychological-possession condition* (participants possessed a coupon, which can be redeemed for a placebo analgesic oil, inducing an expectation to acquire analgesic benefit later upon the experience of pain), *or no-possession condition*. Participants did a cold pressor test (CPT) to experience experimentally-induced pain on their non-dominant hand. Their objective physical pain responses (pain-threshold and pain-tolerance), and subjective psychological pain perception (pain intensity, severity, quality, and unpleasantness) were measured. Results revealed that participants in the physical-possession condition reported greater pain-threshold, *F*(2, 85) = 6.65, *p* = 0.002, and longer pain-tolerance, *F*(2, 85) = 7.19, *p* = 0.001 than participants in the psychological-possession and no-possession conditions. No significant group difference was found in subjective pain perception. The results of this study can advance knowledge about pain mechanisms and novel pain management.

## Introduction

A placebo response occurs when one experiences psychological and/or physical changes upon using medical treatment procedures or substances that inherently have no curing power^1,2^. A great deal of research has investigated the placebo effect in the area of pain and analgesia. These prior studies on placebo analgesia usually involved the *use* of a placebo analgesic and provided evidence suggesting the existence of placebo analgesic responses^1–5^. Although using a placebo analgesic first requires possessing it, the impact of merely possessing a placebo analgesic has been largely neglected in the research literature. Recently, it has been demonstrated that, under certain circumstances, *merely possessing* (without using) a placebo analgesic is sufficient to reduce pain; participants merely possessing a placebo analgesic showed improved placebo analgesia when compared to participants without possessing a placebo analgesic^6–8^. In these studies, mere possession occurred when participants were given a tangible placebo analgesic cream directly and immediately during the experiment. Such physical possession creates an expectation to acquire the analgesic benefit early upon the experience of pain. However, possession can also be psychological in that people can hold a perception or feeling or knowledge of possessing an analgesic; for instance, possessing an analgesic prescription. This form of possession creates an expectation to acquire the analgesic benefit later, if desired. In this paper, we report a randomized controlled experiment examining how temporal expectancy (expecting to acquire analgesic benefit earlier vs. later) implicated by different forms of analgesic possession (physical vs. psychological possession) affects people’s objective physical pain responses and subjective psychological pain perception. A control condition in which participants did not possess any analgesic was also included.

Ample psychological research has revealed that individuals experience psychological and cognitive consequences by merely possessing an object. For instance, merely possessing an object is associated with changes in the perception of the object per se^9–15^, and the owners’ domain specific self-efficacy^16–20^. The mere possession effect has also been demonstrated in relation to analgesia. In three recent studies^6–8^, researchers found that when people merely possess (vs. not possess) a tangible placebo analgesic cream, they reported experiencing less pain during a subsequent cold pressor test (CPT). For example, Yeung et al. either provided or did not provide participants with a placebo analgesic cream^6^. When subjected to a CPT, participants who possessed the placebo analgesic cream reported a lower perception of pain when compared to participants who did not possess the cream. Consistently, Yeung et al. allowed participants to either apply or possess a placebo analgesic cream, possess a pain-irrelevant cream (described as not treating pain), or no cream^7^. Participants who possessed the placebo analgesic cream showed better pain outcomes during a CPT than did participants in the pain-irrelevant cream or no cream conditions. Interestingly, there were no significant differences in pain outcomes between participants who applied or possessed the analgesic cream. Yeung et al.^7^ speculated that this possession-based placebo effect was driven by participants’ expectation that simply possessing the analgesic will transfer the benefit to the self (i.e., the object-to-self transference expectancy^19,20^). Specifically, Yeung et al.^7^ found that when participants possessed (but did not use) a placebo analgesic cream and were asked to anticipate how the placebo analgesic cream benefited the self (i.e., underwent the object-to-self transference expectancy), their performance was comparable to participants who actually *used* the placebo analgesic (Study 1a); and such mere possession effect was weakened once participants were not allowed to undergo the object-to-self transference expectancy (Study 1b)^7^. Lastly, Yeung and Geers replicated and further elaborated the possession-based placebo effect^8^. They found that the possession effect occurred when participants had no immediate prior pain exposure, but the effect disappeared when participants were exposed to a pain stimulus prior to a CPT. The researchers suggested that when participants have no prior pain exposure, it was easier to form “the object-to-self transference expectancy”, which led to placebo analgesia. It should be noted that all these past studies examined the analgesic effect of ownership of a *tangible* placebo analgesic cream (i.e., physical possession).

Ownership generally refers to the state of possessing and having dominate control over an external object. It describes a relationship between the owner and the owned object; such a relationship could be very strong as people treat their possession as an extension of the self^9,21^. Ownership can take two different forms. First, the actual physical possession and dominance over a tangible object. Second, the psychological perception or feeling that one owns and dominates a concrete object or abstract concept (e.g., ideas, investments, a job, or an organization)^22^. In prior possession-based placebo studies^6–8^, the possession predominantly took the form of physical ownership—participants were given possession of a tangible placebo analgesic cream. However, in reality, patients may not actually possess medication immediately after consulting a doctor and instead are provided with a prescription (especially in Western clinical practice), which can later be redeemed for the medication. This constitutes a psychological possession of medication. While physical possession implies acquiring the treatment effect quickly, psychological possession implies the delayed acquisition of the treatment effect. Based on the above, the current study investigated the impact that form of possession (physical versus psychological) of an analgesic had on pain outcomes.

We argue that it is not just possessing and using a placebo analgesic that affects perception of pain; participant beliefs about the speed at which the analgesic benefits can be experienced also influences their pain outcomes. For example, two recent studies revealed that people holding an expectation that the placebo analgesic cream has an early (vs. late) treatment onset time point reported experiencing greater placebo analgesia^23,24^. In the first study, participants applied an (inert) analgesic cream on their arm and were then administered a painful shock via a somatosensory stimulator^23^. Participants were informed (prior to the shock) that the analgesic effects of the cream would be experienced at various time points (5-min vs. 15-min vs. 30-min) after application. Results showed that participants who expected the cream to have prompt effect after application (5-min group) showed an early analgesic effect (decreased pain intensity) compared to the other two groups. In the second study, pain was induced via a CPT, and participants were informed that the (inert) analgesic cream would take effect 5- or 30-min after application^24^. Results showed that, 10 min after application of the cream, placebo analgesia (increased pain tolerance) occurred only in those participants who expected an early onset of analgesia (i.e., in the 5-min condition).

Based on the evidence that anticipation of quicker analgesic effects of a placebo cream results in a reduced experience of pain, it was hypothesized that physical (vs. psychological) possession of a placebo analgesic would show a stronger placebo analgesia effect as it implied an early (vs. late) acquisition of the treatment benefit.

## Method

### Overview

Following past studies^6–8^, participants were first shown a placebo analgesic. Their exposure, accessibility, and interaction with the presented placebo analgesic were kept constant (they inspected the analgesic for 1 min but not allowed to touch or use it). In the current study, all participants were instructed to think about how the presented analgesic would benefit them (i.e., underwent the object-to-self transference expectancy). Then, some participants received a tangible placebo analgesic (physical-possession condition); some received a coupon to be redeemed for a placebo analgesic (psychological-possession condition); and some did not receive any placebo analgesic (no-possession condition). Participants performed a CPT to experience an experimentally-induced pain on their non-dominant hand. Their physical pain responses were objectively assessed by pain threshold (how fast the skin registers an initial physical pain sensation) and pain tolerance (how long the hand can endure the physical pain). Their psychological pain responses were subjectively assessed via self-reported ratings on pain intensity, severity, quality, and unpleasantness (completed after exposure to the CPT). Everything was identical across the three conditions except for the possession status (physical vs. psychological vs. no), as participants were randomly assigned to different conditions, any subsequent group differences in the pain outcomes thus shall be attributed to the differential possession status.

While prior possession-based placebo studies ^6–8^ used a placebo analgesic cream as a stimulus object, the present study adopted a new stimulus object, a vial of placebo analgesic essential oil, to examine generalizability of findings. Past studies showed that essential oil or aromatherapy is an alternative and complementary treatment that can effectively help clinically diagnosed medical ailments^25,26^. Participants across the three conditions were introduced to a vial of (placebo) analgesic essential oil (in fact, olive oil), which was used intranasally. Participants who were in the possession group were not permitted to use or inhale the oil to create a mere possession status. Participants’ emotional states (anxiety and affect), expectancies (expect the presented analgesic oil is effective, expect the self is able to handle the pain, and expect the CPT is of low pain intensity and severity), motive, social desirability as well as personality variables that might affect placebo analgesia^27,28^ were also measured (justifications of assessing these variables are given in the “Supplementary Materials”).

### Participants

The results of a G-power analysis (based on a previous experiment^6^), indicated that a sample of 90 participants (power = 0.90, α = 0.05 and $${\eta }_{p}^{2}$$ > 0.13) was necessary for this study. The research obtained ethical approval from Lingnan University Research Ethics Committee, and all methods were carried out in accordance with ethical guidelines for the treatment of human participants. Informed consent was obtained from all participants. Recruitment was conducted via an email invitation and flyers distributed on the university campus between May and August 2019. Interested students registered on a Qualtrics platform and indicated that they met inclusion criteria (consistent with several previous studies^29–33^) including not being: pregnant, a current smoker, injured on the non-dominant hand, allergic to essential oils, hypertensive, diabetic, in chronic pain. In addition, participants must not have a chronic kidney disease, a circulatory disorder, a previous cold injury, a history of cardiac events, seizures, fainting, drug or alcohol abuse, currently use blood pressure medication, opioids, tranquillizers, antidepressants, hormones, or oral contraceptives. Participants also confirmed if their employment, if any, required them to be exposed to extreme hot or cold environment, which may affect pain sensitivity^34^. After screening and obtaining written consent, 90 eligible healthy students (59 females, *M*_age_ = 19.59, *SD* = 1.86) were randomly assigned into one of three possession conditions: physical-possession condition (*n* = 30), psychological-possession condition (*n* = 30) and no-possession condition (*n* = 30). Participants received a remuneration of HK$100 (approximately US$13) for participating in the study.

### Procedure

**Figure 1:**
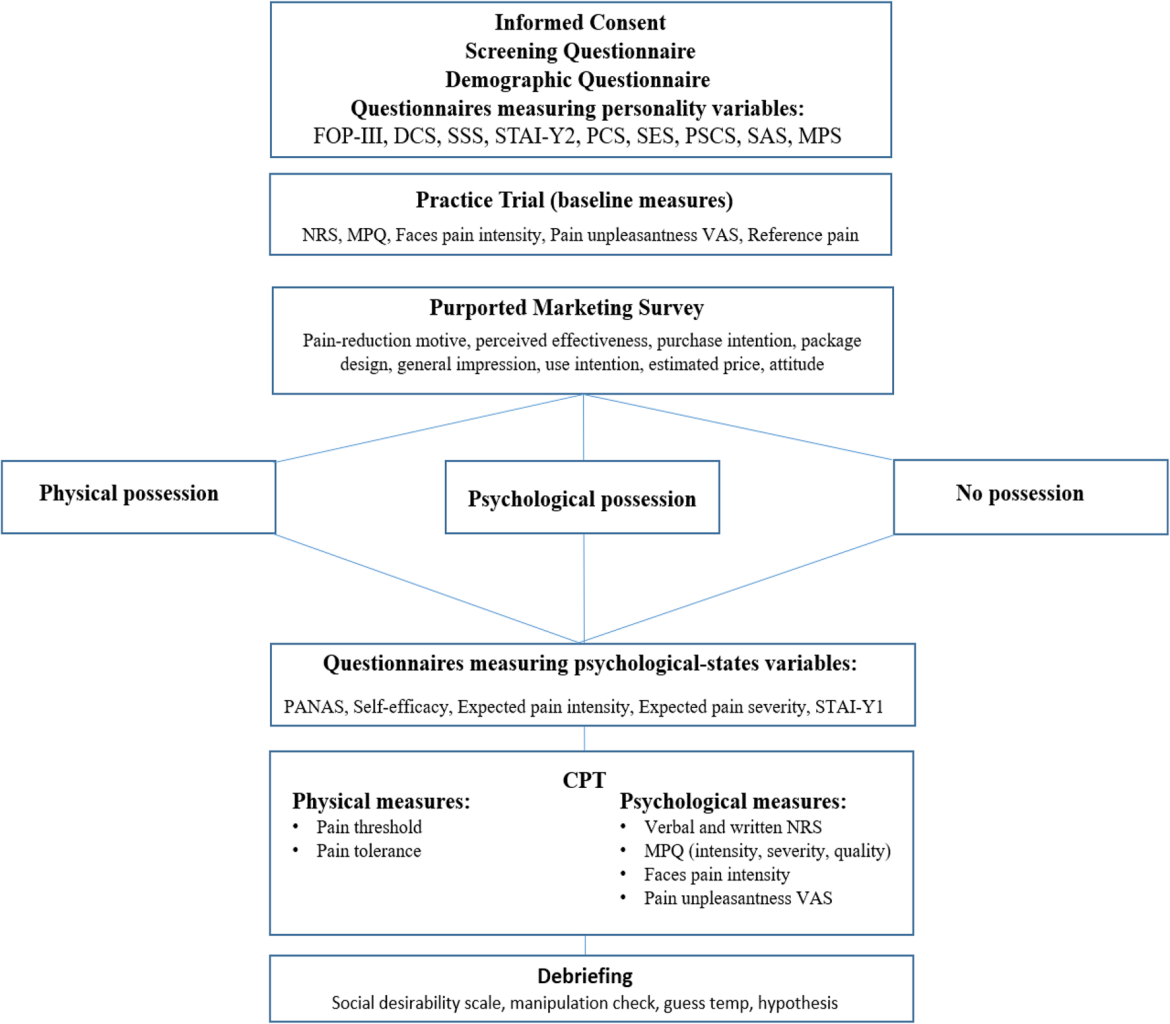
Flowchart of the experimental procedures.

The study procedure is presented in the flowchart diagram in Fig. 1.

#### Cover story

Participants were informed that the study was intended to investigate the relationship between personality and pain perception in Asians. They were told that the study was divided into two parts; in the first part, participants were asked to complete an online personality questionnaire; and, in the second part, they would complete a cold pressor test (CPT) in the laboratory to measure their pain perception.

#### Online personality questionnaire

Participants indicated their consent by pressing “Agree” and declared to provide their best and honest answers. The questionnaire consisted of demographic and personality questions (see “Supplementary Materials”).

#### Laboratory session

One week later, participants attended the laboratory session. They were greeted by a male experimenter in a white lab coat. Participants gave a written informed consent, and confirmed having no recent injury on their non-dominant hand, and no nasal congestion problem on the day of the experiment. Participants were told that a (fictitious) French essential oil company collaborated with a pain research center in the United States and had successfully invented a new essential oil formula that can effectively reduce physical pain via inhalation. Furthermore, participants were told that the manufacturer of this new analgesic essential oil intended to expand its market to Asia. Participants were invited to participate in order to assist the manufacturer in adjusting the active ingredients of the oil to Asian population. Participants were asked to complete two tasks: first, a CPT to measure their pain perception; and second, a marketing survey to give their opinion on the new analgesic essential oil product from the perspective of Asian consumers. It should be noted that in order to minimize any potential experimenter effects, the experimenter was blinded to the study hypotheses, and a written script was used throughout the experiment to standardize the experimenter–participant interactions.

### Inducing a pain-reduction motive

Participants were asked to read an information sheet to learn some facts about pain prior to doing a CPT. In fact, the information sheet was used to induce participants with a pain-reduction motive. On the information sheet, participants read fictitious information describing the disadvantages of experiencing pain and the advantages of reducing pain. Afterwards, they completed a comprehension test consisting of seven questions to ensure that the information provided were encoded. All participants answered the questions correctly. Participants’ pain reduction motive was assessed by three items (e.g., “*When you experience physical pain, will you try to reduce it?*”; 1 = “Absolutely not”, 7 = “Absolutely yes”). A mean score of pain reduction motive was calculated (Cronbach’s α = 0.81).

### A practice trial of a CPT to obtain baseline data

Participants read an instruction paper to learn about CPT. The instruction was also verbally delivered by the experimenter. Upon confirmation of their understanding of the procedure, participants completed a practice trial of a CPT to become familiar with the procedure and learn to use the various scales to report their pain sensations. The purpose of the practice is to obtain participants’ baseline pain data. The CPT practice trial consisted of participants being presented with an empty container and told to imagine that it was filled with water. They learned the proper way to position their non-dominant hand inside the empty container, and were reminded to hold their hand in the same position during the main CPT. They also learned to report their pain perception using various measurement scales (i.e., verbal Numerical Rating Scale [NRS] every 15 s for three times, written NRS, Faces Pain Scale-Revised, the short form of the McGill pain questionnaire [SF-MPQ], and pain unpleasantness visual analog scale [VAS]), which are described in detail below). After the practice session, participants rested to stabilize their blood pressure and heart rates prior to the main CPT. During the resting period, participants completed the (purported) marketing survey in a quiet space.

### Purported marketing survey

The purported marketing survey was introduced between the practice and main trials of the CPT. The aim is to introduce participants an analgesic (placebo) essential oil. Participants first read some general introductory information about aromatherapy and how it works. They then read a number of research and newspaper articles documenting the usefulness of inhaling essential oil in easing physical pain (these materials were modified based on real newspaper scripts and past research studies^35–42^). Participants needed to answer three questions correctly to indicate their understanding of the materials before proceeding to the next step. All participants provided correct answers. Participants were then presented with a professionally designed, color-printed pamphlet promoting a branded analgesic essential oil. The promotional pamphlet aimed to instill in participants a high expectation of the effectiveness of the oil in treating pain. The pamphlet contained fictitious information about the active ingredients, its adoption of advanced nanotechnology and pain blocking mechanisms, its effectiveness, use, and storage guidelines. Participants then completed eight multiple choice questions to check their understanding of the pamphlet’s content and the product. All participants answered all items correctly.

In the next phase of the study, participants were visually presented with a 20 ml bottle of the branded analgesic essential oil. The product was placed inside a transparent display box (9.5 cm × 7.5 cm × 7.5 cm) so that participants were not able to touch or use it. However, they were given 1 min to inspect the product. Then, participants’ perceived effectiveness of the branded analgesic essential oil was measured (7 items, α = 0.92; e.g., “I estimate the pain reduction power of the branded analgesic essential oil is…”, 1 = very weak, 7 = very strong). In order to mask the purpose of the study by making it resemble a marketing survey, participants were told to indicate their purchase intention (1 item) and use intention (1 item) of the product if it was available in the Asian market. They also evaluated the packaging design (1 item) and overall impression (1 item) of the product. Participants were asked to estimate a market price for the product (1 item), and to indicate their general attitude towards essential oils and analgesic products along the other daily commodities (e.g., moisturizing lotion, antiperspirants). Finally, participants were asked to suggest ways to promote the product in the Asian market. Upon completion of the marketing survey, participants were presented with a sample of the branded analgesic essential oil (5 ml). In fact, the sample was generic olive oil (placebo) that was presented in a brown colored vial (diameter = 2.2 cm, height = 6 cm). A label on the body of the vial indicated the brand, name, and function of the product. The vial together with a piece of instruction paper describing the use directions were further placed inside a transparent plastic bag (9 cm × 12.5 cm). Participants were asked to anticipate three benefits of the analgesic essential oil to the self by writing (this is to induce the object-to-self transference expectancy). Participants were then randomly assigned to one of three possession (i.e., physical possession, psychological possession, or no possession) conditions by the experimenter using a random number generator.

### Randomized conditions

#### Physical-possession condition

Participants were informed that in order to thank them for completing the marketing survey, they were given a sample of the branded analgesic essential oil (5 ml) as a token of appreciation. They signed a receipt acknowledging ownership of the oil. They were reminded not to open the vial to avoid spillage as this may contaminate the room.

#### Psychological-possession condition

Participants were informed that in order to thank them for completing the marketing survey, they were given a coupon for a sample of the branded analgesic essential oil, which could be redeemed at a later time (i.e., 2 weeks after their experiment date). They signed a receipt acknowledging that they were provided the coupon.

#### No-possession condition

Participants were verbally thanked for completing the marketing survey but were not provided with a sample of the oil or a coupon they could use to redeem for a sample of the oil.

It should be noted that across the three possession conditions, participants were equally exposed to the branded analgesic essential oil sample and anticipated its benefits, they only differed in the possession status which created a different expected latency of obtaining benefit from the oil.

### Psychological states after randomization

All participants then completed a questionnaire assessing their psychological states, including the affective states, momentary anxiety, and expectancies (see “Materials” section). After completion, participants moved back to the original space to conduct the main CPT.

### Cold pressor test (CPT)

Participants were instructed to review the CPT procedures again. When participants were ready, they immersed their non-dominant hand into a container containing water at room temperature (*M* = 22.17 °C, *SD* = 0.74) for 1 min to standardize the initial skin temperature across participants. Then, they submerged the same hand into a cooling thermostat (Model: Lauda RA12, Bath opening: 300 × 190 × 160 mm, Temperature control: ± 0.05 °C) containing cold water at 5 °C. Participants were not informed the water temperature. Their objective physical responses (pain threshold and pain tolerance), and subjective self-reported responses (NRS, MPQ, Face Pain, Pain unpleasantness) were assessed by the following methods:

#### Objective physical measures

Participants were instructed to ring a bell when they first felt physical pain sensation on their skin. They were reminded to keep their hand submerged in the water for as long as they can, and only pull out their hand when they could no longer tolerate the pain. A timer was used to measure the time between hand immersion and bell ringing (pain threshold), and between hand immersion and hand withdrawal (pain tolerance). Pain threshold indicates how fast participants sense an initial physical pain sensation on their skin. Pain tolerance indicates how long participants can endure physical pain.

#### Subjective self-reported measures

When participants’ hands were still submerged in the water, they verbally reported a number from 0 = “No pain at all”, to 10 = “Extreme Pain” to indicate their subjective perception of pain intensity. Participants reported a number at 15 s intervals until they could no longer withstand the pain or until 3 min had passed, whichever was earlier (for safety reason, the maximum immersion duration was set at 3 min following previous studies^6–8^). After hand withdrawal, participants completed a questionnaire measuring their subjective feelings about pain intensity, severity, quality, and unpleasantness (see “Materials”).

Finally, participants’ tendency to provide socially desirable answers was assessed by the Marlowe–Crowne Social Desirability Scale^43^ (Cronbach’s α = 0.72). Participants were asked to guess the hypothesis of the study and to estimate the temperature of the cold water. They indicated whether they received a gift and what it was (manipulation checks). Participants were thoroughly debriefed at the completion of the study both verbally and in written form.

### Ethical approval

The research obtained ethical approval from Lingnan University Research Ethics Committee, and all methods were carried out in accordance with ethical guidelines for the treatment of human participants.

### Informed consent

Informed consent was obtained from all participants.

## Materials

All materials and instruments were in Chinese. Established Chinese scales were adopted, and if unavailable, English scales were translated to Chinese with procedures of back-translation to ensure linguistic equivalency. The personality scales used in the online survey are presented in “Supplementary Materials”. Below are descriptions of psychological-state scales (measuring affect, self-efficacy, expected pain intensity and severity, and state anxiety) used before CPT; and pain scales (NRS, MPQ, face pain scale, unpleasantness VAS) used during and after CPT.

### Scales assessing psychological states

#### Positive and negative affects schedule (PANAS) scale^44^

The PANAS assesses participants’ mood after they were assigned to different possession conditions. The scale consists of 20 adjectives describing positive (10 items, e.g., “*Enthusiastic*”) and negative (10 items, e.g., “*Nervous*”) mood states. Participants rated the extent to which they felt the described mood (1 = “Very Slightly or Not at All”, 5 = “Extremely”). Mean scores of positive (α = 0.88) and negative (α = 0.85) mood states were calculated.

#### *Self-efficacy of pain resilience scale*^7^

This 16-item scale assesses participants’ perceived self-efficacy to manage pain from a CPT (e.g., “*During the CPT, I could manage any discomfort*”; 1 = “Strongly disagree”, 7 = “Strongly agree*”*). The mean score of perceived self-efficacy was calculated (α = 0.94).

#### *Expected pain intensity item*^45^

An item (“*How much pain do you expect during the cold pressor test?*”) was included to assess participants’ expected pain intensity (0 = “No Pain at All”, 5 = “Moderate Pain”, to 10 = “Extreme Pain”).

#### *Expected pain severity item*^46^

An item (“*What would be the severity of pain you expect to experience when you put your hand into the cold water?*”) was included to measure participants’ expected pain severity (1 = “No Pain at All”, to 5 = “Excruciating”).

#### *State-anxiety scale*^47^

Participants’ state anxiety level prior to the main CPT was assessed using the 20-item State-Trait Anxiety Inventory (STAI-Y1). The STAI-YI measures the presence (10 items, e.g., “*I am tense*.”) and absence (10 items, e.g., “*I feel calm*.”) of anxiety. Using a four-point Likert scale, participants indicated the extent to which they were feeling anxious (1 = “Not at all”, 4 = “Very much so”). The anxiety absent items were reversed scored. The mean score of state anxiety was calculated (α = 0.90).

### Subjective self-reported pain measures

#### *Numerical rating scale (NRS)*^48^

The NRS was adapted to measure real time (verbal) and retrospective (written) pain intensity. With respect to real-time pain intensity, participants were shown an instruction paper printed with a 11-point scale (from 0 = “No Pain at all” to 10 = “Extreme Pain”). During the CPT, participants verbally reported a number every 15 s corresponding to their level of pain intensity. Participants terminated the CPT when they could no longer endure the pain or when 3 min had passed, whichever was earlier. Participants used the same scale and wrote down a number indicating their pain intensity right after the CPT, thus measuring retrospective pain intensity.

#### *Short form of McGill Pain Questionnaire (SF-MPQ)*^46^

The SF-MPQ measures participants’ perceived pain intensity, severity, and quality upon hand withdrawal. In the pain intensity part, participants selected one of the six descriptors (0 = “No Pain”, 1 = “Mild”, 2 = “Discomforting”, 3 = “Distressing”, 4 = “Horrible”, and 5 = “Excruciating”) to depict their current, least, and worst pain perceived in the CPT (α = 0.68). The same rating scale was also used to depict their worst toothache, headache, and stomachache, the mean score of which provides a reference point of pain intensity (α = 0.57). In the pain severity part, participants marked a cross on a 10 cm long horizontal line (with a label of “No Pain at All” printed on the left end and “Extreme Pain” printed on the right end). The distance between “No Pain at All” and the marked cross indicated the perceived pain severity. In the pain quality measure, participants rated fifteen adjectives associated with the experience of pain (e.g., “*Stabbing*”) using a four-point scale (0 = “None”, 1 = “Mild”, 2 = “Moderate”, and 3 = “Severe”). The mean score of perceived pain quality was calculated (α = 0.87).

#### *Faces pain scale-revised*^49^

Participants were presented with a scale which consisting of six different faces representing an increasing degree of pain intensity (far left face: 1 = “No Pain”, far right face: 6 = “Very much pain”). Participants circled a face to represent their perceived pain intensity during administration of the CPT.

#### *Pain unpleasantness visual analog scale (VAS)*^50^

The VAS measures the degree of pain unpleasantness participants felt from the CPT. Participants marked a cross on a 10 cm long horizontal line (left end point = “Not unpleasant at all”, right end point = “Extremely unpleasant”). The distance between the left end point and the marked cross indicated participants’ unpleasant feeling. The longer the distance (in cm), the more unpleasant feeling the participants experienced.

### Statistical analysis

SPSS 23 was used for data analysis. Tables 1 and 2 show the means, *SD*s, and *F*-statistics for each of the dependent measures in each possession condition. One-way ANOVAs were conducted with possession condition (physical vs. psychological vs. no) as the independent variable, and each pain outcome as the dependent variable. When a significant omnibus *F*-test was found, the post-hoc comparisons were conducted to compare how the possession conditions significantly differed from each other. The Tukey’s honest significant difference test was used if the assumption of homogeneity of variance was satisfied; otherwise, the Games-Howell post-hoc test was used. Data normality was assessed by the Shapiro–Wilk test. If normality was violated, the Welch’s *F* test was performed, and the omega squared was reported. The significance level was set at α = 0.05 (two-tailed test).

**Table 1:** Participants’ demographics in each possession condition.

	Physical-possession (n = 30)	Psychological-possession (n = 29)	No-possession (n = 29)
Gender (male/female)	12/18	10/19	8/21
Mean age (*SD*)	20.48 (2.44)	18.83 (0.97)	19.55 (1.53)

**Table 2:** The means, standard deviations (in parentheses), and F-statistics of psychological-state variables as a function of possession condition.

	Scale	Physical-possession (*n* = 30)	Psychological-possession (*n* = 29)	No possession (*n* = 29)	*F*-statistics^a^	Effect size *(*${\eta }_{p}^{2})$	Post-Hoc
Pain reduction motive	1–7	5.73 (0.94)	5.75 (0.88)	5.66 (1.05)	0.08	0.002	
Perceived effectiveness	1–7	4.90 (0.86)	5.03 (0.880)	5.03 (0.83)	0.22	0.005	
Guess temperature	°C	− 2.13 (9.00)	− 3.59 (8.95)	− 2.03 (14.71)	0.17	0.004	
Positive affect	1–5	2.27 (0.67)	2.1 (0.67)	2.34 (0.82)	0.66	0.02	
Negative affect	1–5	1.40 (0.47)	1.39 (0.40)	1.40 (0.48)	0.004	0.00	
Perceived efficacy	1–7	4.98 (0.82)	4.72(0.87)	4.80 (0.85)	0.74	0.02	
Expected pain intensity	0–10	6.73 (1.05)	6.00 (1.79)	6.52 (1.50)	1.91	0.04	
Expected pain severity	1–5	2.73 (0.785)	2.48 (0.738)	2.93 (0.753)	2.54	0.06	
State anxiety	1–4	2.14 (0.482)	2.28 (0.457)	2.19 (0.38)	0.79	0.02	
Social desirability	0–13 (yes/no)	5.60 (3.07)	7.34 (2.42)	7.38 (2.87)	3.90*	0.08	Phy < Psy Phy < No Psy = No

Values outside parentheses are means, inside are standard deviations.

*Phy* physical possession, *Psy* psychological possession, *No* no possession.

*p < 0.05.

^a^For univariate *F*-tests, degrees of freedom are (2, 85).

## Results

### Manipulation checks and screening

All participants indicated their possession status correctly. No participants were able to correctly guess the hypothesis of the study; however, two participants later revealed that they had participated in a similar study before, thus their data was discarded, leaving 88 participants for data analysis (physical-possession condition: *n* = 30; psychological-possession condition: *n* = 29; no-possession condition: *n* = 29). Table 1 presents participants’ demographics across the three possession conditions. Table 2 shows that, in general, all participants held a high pain-reduction motive (*M* = 5.71, *SD* = 0.95), viewed the presented essential (olive) oil as an effective inhaling analgesic (*M* = 4.99, *SD* = 0.85), *p*s = *ns*, and overestimated the coldness of the water (guessed temperature: *M* = − 2.61 °C, *SD* = 11.09). Participants’ performance in the personality scales and marketing survey did not differ based on their possession condition. Findings of their personality variables and marketing survey responses in relation to the pain outcomes are reported in “Supplementary Materials”.

### Baseline pain measures

One-way ANOVAs were performed to consider any pre-existing group differences in the baseline pain measures prior to the main CPT. Table 3 shows that during the CPT practice trial, all participants rated their pain level to be “0”, indicating no initial pain feeling. There were no pre-group differences on NRS, MPQ pain intensity and severity, face pain intensity, and unpleasantness VAS ratings. No group difference was found in the reference pain from natural sources (toothache, headache, stomachache; *M* = 3.12, *SD* = 0.84), implying no tendency to exaggerate/understate feelings of pain. Overall, participants in the three possession conditions did not differ in all baseline pain measures.

**Table 3:** The means, standard deviations, (in parentheses) and F-statistics of pain outcomes as a function of possession condition.

	Scales	Physical-possession (*n* = 30)	Psychological-possession (*n* = 29)	No-possession (*n* = 29)	*F*-statistics^a^	Effect size (${\eta }_{p}^{2})$	Post-Hoc^b^
**Baseline measures**							
Verbal and written pain intensity NRS	0–10	0.00 (0.00)	0.00 (0.00)	0.00 (0.00)			
MPQ pain intensity	0–5	0.00 (0.00)	0.00 (0.00)	0.00 (0.00)			
MPQ pain severity	cm	0.00 (0.00)	0.00 (0.00)	0.00 (0.00)			
Face pain intensity	1–6	0.00 (0.00)	0.00 (0.00)	0.00 (0.00)			
Pain unpleasantness VAS	cm	0.16 (0.86)	0.00 (0.00)	0.01 (0.04)	0.90	0.02	
Reference pain	0–5	3.31 (0.91)	3.10 (0.86)	2.94 (0.72)	1.43	0.03	
**Objective physical measures**							
Pain threshold	s	14.77 (14.23)	7.62 (4.62)	7.20 (3.73)	6.65**	0.14	Phy < Psy Phy < No Psy = No
Pain tolerance	s	130.37 (66.88)	69.51 (54.19)	88.89 (67.11)	7.19**	0.15	Phy < Psy Phy < No Psy = No
**Subjective self-reported measures**							
Verbal pain intensity NRS	0–10	7.08 (1.37)	6.58 (1.41)^c^	6.74 (1.13)^d^	1.07^e^	0.03	
Written pain intensity NRS	0–10	8.10 (1.71)	8.38 (1.76)	7.90 (2.02)	0.51	0.01	
MPQ pain intensity	0–5	2.98 (0.87)	3.02 (0.84)	3.01 (0.96)	0.02	0.000	
MPQ pain severity	cm	7.46 (1.79)	7.73 (1.94)	7.30 (2.11)	0.36	0.009	
MPQ pain quality	0–3	1.29 (0.53)	1.40 (0.61)	1.37 (0.54)	0.34	0.008	
Face pain intensity	1–6	3.37 (1.10)	3.41 (1.21)	3.41 (1.05)	0.02	0.00	
Pain unpleasantness VAS	cm	5.75 (2.72)	6.11 (2.55)	6.05 (2.98)	0.15	0.003	

Values outside parentheses are means, inside are standard deviations.

*Phy* physical possession, *Psy* psychological possession, *No* no possession.

**p < 0.005.

^a^For univariate *F*-tests, degrees of freedom are (2, 85).

^b^Games–Howell post-hoc comparisons were utilized.

^c^*n* = 27.

^d^*n* = 26.

^e^For univariate *F*-tests, degrees of freedom are (2, 80).

### Objective physical responses

**Figure 2:**
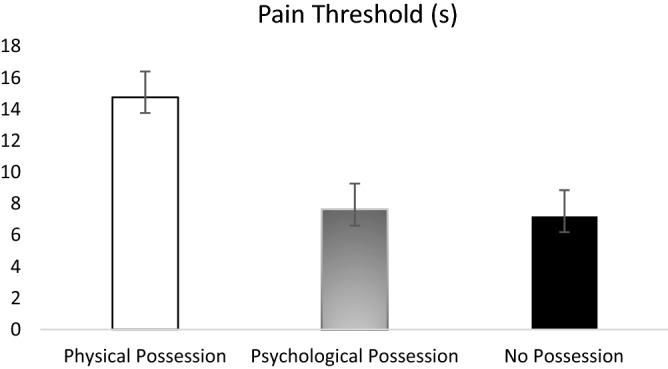
Main effect of possession condition on pain threshold. Participants in physical possession condition took longer to feel an initial pain sensation than participants in psychological possession condition and no possession condition. Error bars show 95% confidence intervals.

Participants’ physical pain responses were assessed by pain threshold (how fast they detected an initial pain sensation) and pain tolerance (how long they could endure the pain). First, a one-way ANOVA was conducted with possession condition as the independent variable and pain threshold as the dependent variable. The main effect of possession condition on pain threshold was significant, *F*(2, 85) = 6.65, *p* = 0.002, ${\eta }_{p}^{2}=0.14$, observed power = 0.90, CI_90%_ [0.03, 0.24]. The *Levene’s F* test revealed that the homogeneity of variance assumption was not met, *F*(2, 85) = 8.34, *p* < 0.001; and Shapiro–Wilk test revealed that the data distribution was not normal, *W*(88) = 0.66, *p* < 0.001. As such, the *Welch’s F* test was used. The one-way ANOVA of participants’ average threshold by possession condition revealed a significant main effect, *Welch’s F*(2, 85) = 6.65, *p* = 0.002, indicating that participants across the three possession conditions did differ significantly in sensing the initial physical pain. The estimated omega squared, ω^2^ = 0.11, indicated that approximately 11% of the total variation in pain threshold was attributable to differences between possession conditions. Games-Howell post hoc comparisons were thus conducted to determine which pairs of condition differed on pain threshold. Results indicated that participants physically possessing a tangible placebo analgesic oil (*M* = 14.77 s, *SD* = 14.23) took significantly longer to report becoming aware of sensations of pain on their skin than did participants possessing a coupon for a placebo analgesic oil (*M* = 7.62 s, *SD* = 4.62), *p* = 0.03, or participants who have no possession at all (*M* = 7.20 s, *SD* = 3.73), *p* = 0.02. Participants in the psychological-possession condition and no-possession condition did not differ from each other, *p* = 0.92. Figure 2 shows the group differences on pain threshold.

**Figure 3:**
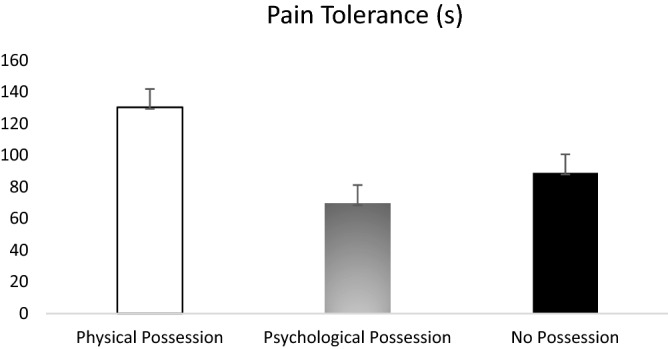
Main effect of possession condition on pain tolerance. Participants in physical possession condition showed longer pain tolerance than participants in psychological possession condition and no possession condition. Error bars show 95% confidence intervals.

Second, a one-way ANOVA was conducted with possession condition as the independent variable and pain tolerance as the dependent variable. A significant main effect of possession condition on pain tolerance was found, *F*(2, 85) = 7.19, *p* = 0.001, ${\eta }_{p}^{2}=0.15$, observed power = 0.93, CI_90%_ [0.04, 25]. *Levene’s F* test revealed that the homogeneity of variance assumption was violated, *F*(2, 85) = 3.25, *p* = 0.04; and Shapiro–Wilk test indicated that the data departed from normality, *W*(88) = 0.81, *p* < 0.001. The *Welch’s F* test was therefore conducted. Result revealed that the main effect of possession condition on pain tolerance was statistically significant, Welch’s *F* (2, 85) = 7.19, *p* = 0.001, ω^2^ = 0.12. Games-Howell post hoc comparisons revealed that participants in the physical-possession condition were able to endure the pain significantly longer (*M* = 130.37 s, *SD* = 66.88) than participants in the psychological-possession condition (*M* = 69.51 s, *SD* = 54.19), *p* = 0.001, and no-possession condition (*M* = 88.89 s, *SD* = 67.11), *p* = 0.05. There was no significant difference between psychological-possession condition and no-possession condition, *p* = 0.45. Figure 3 shows the group differences on pain tolerance.

**Figure 4:**
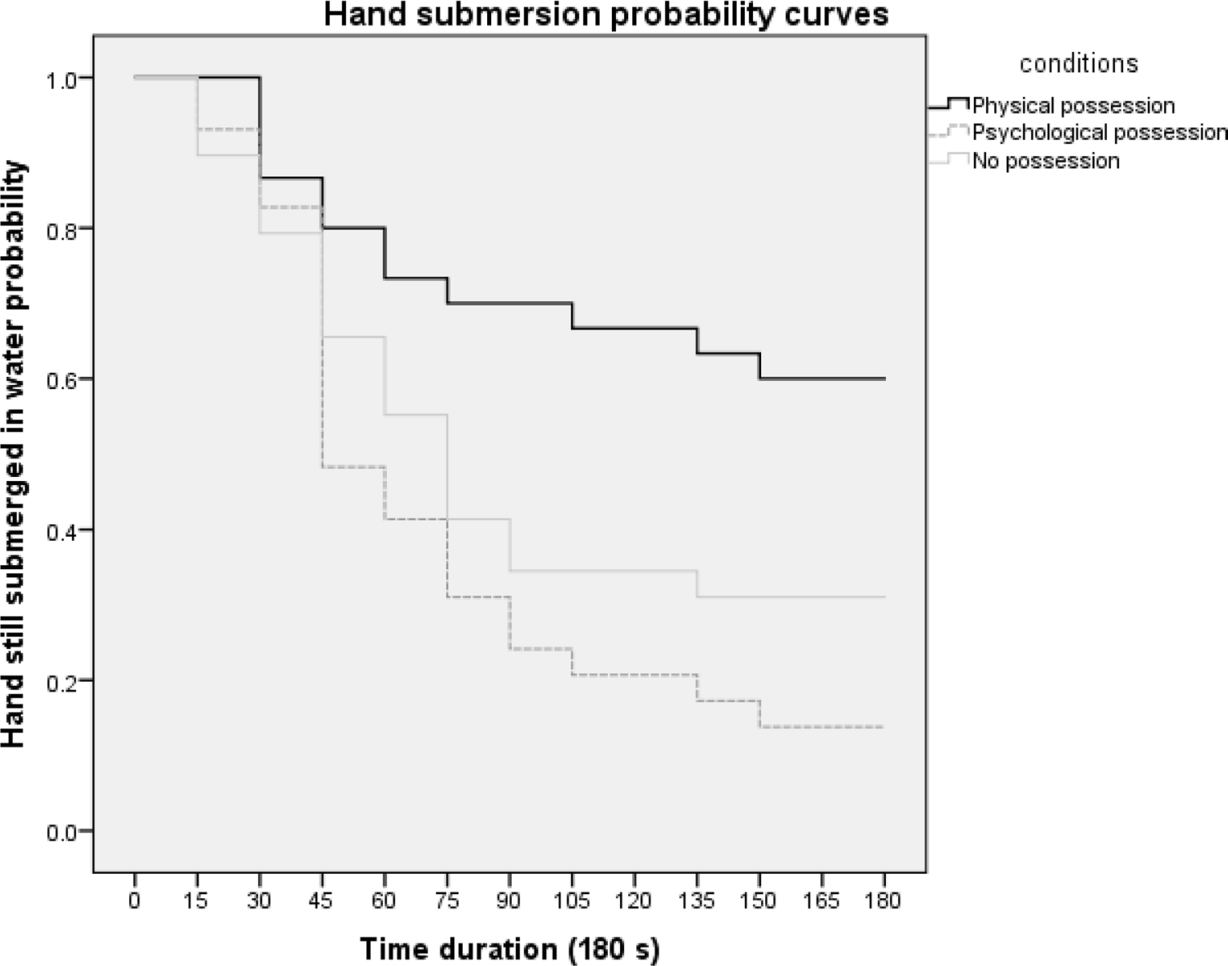
The hand submersion probability curves as a function of time duration for the three possession conditions. Participants physically possessing a placebo analgesic showed a higher probability of keeping their hand submerged in cold water than participants psychologically possessing the placebo analgesic and participants without possessing any placebo analgesic.

Since participants can freely withdraw their hand whenever they choose, a cox regression analysis was conducted taking into consideration the number of participants who withdrew their hand and the timing of hand withdrawal at each 15-s time interval across the entire 180 s immersion time. Omnibus test of model coefficients suggested a significant main effect of possession condition, *X*^2^(2) = 13.17, *p* = 0.001. Participants in the physical-possession condition showed greater probability to persistently submerge their hand in the cold water than participants in the psychological-possession condition, Exp(β) = 3.43, *p* = 0.001, and no-possession condition, Exp(β) = 0.43, *p* = 0.02. There was no significant difference between the psychological-possession condition and no-possession condition, Exp(β) = 0.1.48, *p* = 0.19. Figure 4 shows the hand submersion probability curves as a function of time duration for the three possession conditions, respectively.

### Subjective self-reported responses

Participants’ subjective pain responses were indicated by verbal and written NRS, MPQ pain intensity, severity and quality, face pain intensity, and pain unpleasantness VAS. ANOVAs were conducted with possession condition as the independent variable and each of these self-reported measures as the dependent variable. No significant main effect of possession condition was found on any of the self-reported measures (see Table 3).

### Psychological states

After the manipulation of possession status, but before the administration of the main CPT, participants’ psychological states were measured (see Table 2). One-way ANOVAs were conducted with possession condition as the independent variable and each of the psychological-state measures (positive and negative affect, perceived efficacy, expected pain intensity, expected pain severity, state anxiety, social desirability) as the dependent variables. It was found that participants shared similar level of emotional states regardless of their possession condition (positive affect: *M* = 2.25, *SD* = 0.72; negative affect: *M* = 1.40, *SD* = 0.45; state anxiety: *M* = 2.20, *SD* = 0.44), all *p*s = ns. Participants generally have high expectancies, for instance: expected the self to have high efficacy to tackle pain (*M* = 4.83, *SD* = 0.84), and the CPT to have moderate pain intensity (*M* = 6.42, *SD* = 1.49) and low pain severity (*M* = 2.72, *SD* = 0.77), all *p*s = ns. This is probably due to that all participants were simultaneously instructed to engage in the object-to-self transference expectancy [i.e., an expectancy that the (placebo) analgesic transfers the benefit to the self]. Lastly, given that participants in the physical-possession condition showed a lower social desirability tendency (*M* = 5.60, *SD* = 3.07) than participants in the psychological-possession condition (*M* = 7.34, *SD* = 2.42) and no-possession condition (*M* = 7.38, *SD* = 2.87), *F*(2, 85) = 3.90, *p* = 0.03, ${\eta }_{p}^{2}$ = 0.08, observed power = 0.69, CI_90%_ [0.006, 0.18], social desirability was therefore submitted to simple regression to assess its impact on pain outcomes. Results showed that social desirability did not significant predict any of the pain dependent variables, *ps* = ns.

## Discussion

Although recent research showed that the mere possession of a placebo analgesic enhances placebo analgesia^6–8^, no research has comprehensively investigated the relationship between different *forms* of possession on the effect of placebo analgesia. In the current study, possession of a placebo analgesic was varied; participants were either given a tangible placebo analgesic (physical-possession condition), a coupon for a placebo analgesic (psychological-possession condition), or not given any possession at all (no-possession condition). It was found that participants reported placebo analgesia when they had physical ownership of a placebo analgesic compared to when they possessed symbolic ownership (i.e., a coupon) of a placebo analgesic, or no ownership. The current work identifies a specific condition crucial for possession-induced placebo analgesia.

The following question was raised: why does physical (vs. psychological) ownership better facilitate placebo analgesia? In the current study, it was proposed that physical ownership of a placebo analgesic creates a situation of early access, resulting in an immediate reward. In contrast, psychological ownership results in a delayed reward. The temporal expectancy to acquire immediate reward from physical ownership facilitates placebo analgesia. To elaborate, prior studies revealed that when people acquire ownership of an object, they expect to obtain reward from the owned object^51–53^. This is supported by neuroimaging studies which demonstrated that self-ownership activated the brain areas responsible for signaling positive reward. Consistently, the nucleus accumbens (NAc), which is involved in the processing and encoding of reward expectation, was strongly related to the formation of placebo responses. In the current study, participants were given ownership of a placebo analgesic and instructed to anticipate its benefit to the self. This subsequently activated the reward expectancy process. More importantly, although participants in both the physical-possession and psychological-possession conditions received a placebo analgesic oil as a reward, participants in the physical-possession condition should perceive the reward as more immediate due to its instant availability. On the contrary, participants in the psychological-possession condition should perceive the reward as a delayed one as it takes time to redeem a coupon for the analgesic oil. Consistently, a recent meta-analytic study demonstrated that the presence of immediate (vs. delayed) rewards predicted actual persistence in goal-related activities^54^. In the current study, participants possessing an immediate reward of a tangible placebo analgesic were indeed able to persistently submerge their hand in the cold water far longer than participants possessing a delayed reward. Furthermore, this finding is consistent with previous findings reported by Camerone et al.^23,24^, in which it was shown that participants reported reduced perception of pain when expecting a fast (vs. slow) treatment onset time after use of a placebo analgesic cream. In the current study, the different forms of ownership provide different temporal information and create different expectancies of treatment onset. The physical ownership of a placebo analgesic oil implies an early acquisition of the therapeutic effect, facilitating better pain outcomes. On the other hand, an analgesic oil coupon (psychological possession) implies a delayed acquisition of the said therapeutic effect, hindering the placebo analgesic effect. It should be noted that in the studies conducted by Camerone et al.^23,24^, participants experienced analgesic effect upon positive verbal suggestion of a short onset time, and participants could withhold a positive expectation for as long as 35 min. In contrast, participants in the current study who were assigned to the psychological-possession condition expected to obtain a tangible analgesic oil 2 weeks after the experiment date; it is likely that such a long delay could have hindered the formation of any positive expectancy. Consequently, a placebo analgesic effect in the psychological-possession condition was not observed in the current study. Future research should test the temporal aspect of the possession effect by manipulating the coupon redemption time through varying the expected latency (short vs. long delayed duration) of obtaining the placebo and examining the potential impact on placebo responses. Alternatively, a similar process could be examined by allowing participants to have physical possession of a tangible placebo analgesic but varying the verbal suggestions as to its therapeutic onset time (immediate vs. late).

In the current study, participants’ pain outcomes were assessed via two dimensions. First, the objective physical dimension was assessed through measures of pain threshold and tolerance. This dimension indicates participants’ skin sensitivity and endurance to coldness, which are more difficult to be subjectively controlled by participants, and which data are objectively captured by a timer. Second, the subjective psychological dimension was measured in terms of self-reported pain intensity, severity, quality, and unpleasantness. This dimension indicates participants’ retrospective memory of pain, which is more subjective (by giving a number on rating scales). Our results showed that participants in different possession conditions differed significantly in the objective dimension but not in the subjective dimension. Specifically, participants in the physical possession condition reported better objective pain outcomes (higher pain threshold and longer pain tolerance) than participants in the psychological possession or no possession conditions. However, participants did not differ in their subjective pain outcomes (i.e., there were no condition differences in self-reported pain intensity, severity, quality, and unpleasantness). It is interesting that participants showed different actual behaviors (pain threshold and pain tolerance) but not perceived experience of pain (self-reported pain intensity etc.), especially as the placebo effect is about perception and belief.

There are several possible reasons for this pattern of results. First, cumulative evidence from experimental and clinical studies has suggested that the placebo effect can be explained by expectancy^55–57^. Some frequently mentioned expectancies include the expectancy that the treatment or substance is effective^58,59^, that the self is able to cope with pain^60–63^, and that the pain involved in the task is less intense^45^. Recently, two more expectancies have been proposed, namely, the expectancy that the treatment or substance transfers the benefit to the self (i.e., object-to-self transference expectancy)^6–8^, and the expectancy that the substance has an early onset treatment time (i.e., temporal expectancy)^23,24^. In the design of prior possession-induced placebo studies^7,8^, the induction of temporal expectancy and object-to-self transference expectancy varies depending on the possession status: only participants in the possession condition were given physical ownership of a tangible placebo analgesic cream, and asked to anticipate how the owned analgesic benefits the self. Participants in the no-possession condition did not own any analgesic cream and did not undergo any anticipation process. In such a design, participants in the possession condition thus monopolized both the temporal expectancy and object-to-self transference expectancy, consequently showing positive pain outcomes in both objective and subjective measures^7,8^. Nevertheless, in the current design, participants across all conditions were simultaneously instructed to anticipate how the placebo analgesic oil might bring benefit to the self. In other words, the object-to-self transference expectancy was no longer monopolized by the possession condition but being held constant across all conditions. Our data revealed that after participants in all conditions engaged in the object-to-self transference expectancy, the three groups did not show any significant differences in their subjective pain outcomes—even participants in the no-possession condition could now improve their subjective pain outcomes to the same level as their counterparts in the two possession conditions. In the current design, the only difference among the three groups was in their differential temporal expectancy. The results of the current study were consistent with Camerone et al.^24^. In Camerone et al.’s study using a CPT^24^, their participants were manipulated to have different temporal expectancies (i.e., expected an early vs. delayed analgesic onset time of the applied placebo cream), and they also found a placebo effect only in objective pain measure (pain tolerance), but not in subjective pain measure (e.g., self-reported pain intensity). Future research should systematically explore how different types of expectancies affect subjective and objective pain outcomes, respectively.

Second, the disjunction between the objective and subjective pain outcomes may lie in the methodology used to assess the subjective measures. Specifically, the subjective pain ratings were conducted retrospectively after the CPT, and this approach would attenuate differences in pain ratings. To elaborate, consider a situation where two participants initially do not differ in the amount of pain that they could tolerate (e.g., both can tolerate the pain subjectively with an intensity rating of 5), yet one participant is given a tangible analgesic placebo oil and the other one is not. The participant possessing the tangible analgesic oil will take longer to experience an initial pain sensation (i.e., higher pain threshold), and will be able to tolerate the pain stimulation longer (i.e., longer pain tolerance). This way, the participant will take longer to self-report a subjective pain intensity rating of 5; while the participant without the tangible analgesic placebo oil will withdraw his/her hand earlier and still reporting a subjective pain intensity rating of 5. Based on this, both participants reported the same subjective pain intensity rating of 5; however, the reduced subjective pain that results from mere possession of a tangible placebo is actually reflected in the longer tolerance of pain stimulations, but not in the self-report of the retrospective pain. In order to accurately reflect the subjective pain ratings, future research should consider instructing participants to immerse the hand for the same duration and record the pain intensity across various groups of participants. Indeed, when Yeung et al.^6^ standardized participants’ hand submersion duration, they found a significant group difference in participants’ self-reported pain intensity.

Third, the discrepancy between objective and subjective pain outcomes could also be due to the nature of the placebo object being used. Prior placebo studies used placebo cream, while the current study used placebo oil. People may have a different perception about analgesic cream vs. oil in terms of their perceived effectiveness and thus affects the corresponding objective and subjective pain outcomes.

The present study has several limitations. First, like the past studies^7,8^, participants were only instructed to hold the object-to-self expectancy belief, but were not required to rate their object-to-self expectancy level. Future research should include such a measure as a manipulation check. Second, past research showed that tangible possession enhanced people’s feelings of self-determination^64^; and people with higher self-determination were more likely to strengthen placebo effects^60,65^. It is possible that possession of an analgesic (without use) makes people feel more in control to tackle pain, thus enhancing placebo analgesic effects. However, in the current study, participants’ sense of self-determination and perceived control over the placebo analgesic oil were not measured. Future research should examine the roles play by these two variables. Third, the number of male and female participants were not balanced across the three possession conditions. Although meta-analyses revealed poor evidence of association between gender and placebo responses^66^, and we also conducted follow-up statistical analyses with gender as a covariate showing that gender did not pose a significant impact on the outcomes of the current study, it is advised that future research should include samples with equal numbers of male and female participants to examine a potential gender effect in possession-induced placebo analgesia. Finally, we only used a male experimenter. A recent study^67^ revealed that healthy participants engaging with an experimenter of the opposite gender showed greater placebo hypoalgesia using heat-pain stimuli. Future research could include experimenters of both genders and test the possibility of experimenter-participant gender effect.

There are several important implications of the current research findings. First, our investigation enriches the existing literature of the traditional placebo effect which involves the *use* of a placebo, by revealing that sometimes we do not need to actually *use* the placebo to attain a placebo effect. Specifically, the current study demonstrated that placebo analgesia could be achieved even though participants had not inhaled any placebo analgesic essential oil prior to or after experiencing a painful stimulus. The adoption of an inhaling placebo analgesic oil as a stimulus (vs. a placebo analgesic cream used by most prior studies) also increased the generalizability of research findings.

Furthermore, the current work documented an important yet unexplored aspect of the possession-induced placebo effect, suggesting that not being able to immediately possess a (placebo) analgesic increases people’s vulnerability to handle pain. This finding highlights the importance of temporal expectancy of acquiring treatment effect on optimizing treatment outcomes.

The current study also contributes to innovative pain management in medical care and practice. Although participants in the three possession conditions were equal in terms of demographics, personality, evaluation of analgesic product, and baseline pain level, only those who physically possessed a placebo analgesic oil showed a greater pain threshold and pain endurance compared to those with psychological possession and no possession. The findings not only provide further support to the possession-based placebo effect, but also suggest an innovative pain management strategy. An intervention involving an early possession procedure could conceivably be helpful for alleviating pain or enhancing pain tackling ability. For instance, we can capitalize the ownership effect on analgesia by granting physical ownership of pain killers to dental patients immediately prior to dental treatment to improve pain outcomes. Such intervention is inexpensive, non-invasive and can be feasibly administered. Future research can examine whether the relatively trivial procedure of establishing a sense of physical ownership of analgesics can improve pain outcomes in clinical settings and on clinical samples. Future research can also examine what type of individuals (placebo responders^68^) could benefit most from engaging in such possession intervention procedures, thus providing more individualized effective treatment.

The results of the current study also shed light on methods for evaluating the effectiveness of existing clinical practices. In the current study, participants received either a tangible analgesic (physical-possession condition) or a paper coupon to be redeemed for an analgesic (psychological-possession condition). These two possession conditions simulate two real life clinical practices, respectively: (1) letting patients immediately possess the medication after a doctor’s consultation within the clinic (a practice usually found in Asian societies)^69–71^; and (2) delaying the actual possession of an analgesic by giving out a prescription that allows patients to collect the medication later (a practice usually found in Western societies^69–71^). There are several reasons for the need of the latter practice. First, dispensing medicines requires a lot of careful checking and time, as doctors are paid by the number of patients they see, they will try to assess and prescribe (rather than spending time to dispense) as many patients as possible in the least amount of time. Second, patients may not want to wait to obtain the medicine after doctor’s consultation, and they may like to go to the pharmacy to purchase their medicine due to financial or personal preference. Third, and most importantly, there is a clear separating role of physician (prescribing role) and pharmacist (dispensing role) to avoid any potential conflict of interest. The Western practice (separation of prescribing and dispensing) emphasizes and respects the patients’ right and freedom to choose where to purchase the medicine, which is strongly in contrast to the Eastern practice (mixed prescribing and dispensing). In fact, research investigating the separation vs. combination of prescribing and dispensing procedures showed that the separation policy did not work very well in Asian countries^69,70^. Asians are reported to be less willing to adopt a separation policy^71^, and in general are used to obtaining medication directly from the doctor rather than using a prescription procedure. This might explain why our Asian participants in the current study, who are more familiar with the immediate possession practice, benefited more in the immediate (analgesic) possession condition than in the delayed (coupon) possession condition. The finding should be interpreted in consideration of the potential influence of culture^72^. It would be important to replicate the study using Western participants, who are more familiar with the coupon redemption system (i.e., prescription), to examine a cultural effect, if any. A bi-cultural comparison study could also measure some mediating mechanisms, such as, perceptions of the doctor, and examine if it mediates the observed effect.

The finding also has implications for analgesic users/addicts. Patients who are running low on pain killers might feel anxious and worry that they would have to deal with increased pain if no pain medication is available at hand, or that the pain killers that are available are insufficient and will run out very soon. The stress and anxiety experienced by patients probably are not solely due to the addiction or anxiety associated with experiencing (or delaying the experience of) withdrawal symptoms, etc., but also the lack of an immediate possession of pain killers leads them to ruminate about how to obtain more pills. This rumination causes anxiety and would impair their daily functioning. However, if patients know that pain killers are physically available whenever they need them (by its mere ownership, not necessarily taking them), they will feel less anxious. Indeed, prior studies have shown that administration of a placebo lowered anxiety levels^55^, and low state anxiety levels predicted placebo experience or perception^73,74^. We expected that without actually possessing a tangible analgesic, people may feel that they are vulnerable and worry not being able to endure the upcoming pain. On the contrary, the physical possession of a (placebo) analgesic shall lower people’s worry and anxiousness about the forthcoming pain, inducing placebo analgesia. However, it should be noted that in the current study, we did not observe a significantly higher anxiety level in the no-possession group compared to the possession groups. This is probably because our participants were all healthy university students who do not experience the same worry as those patients with opioid dependence. Future research in this area could focus on clinical samples, such as patients with opioid use disorder, and investigate how the fear of not having a suitable remedy at hand affects their ability to deal with greater pain, and whether the mere ownership of the remedy could alleviate their anxiety and physical pain associated with withdrawal symptoms.

Psychological ownership is an interesting concept. When one expects or imagines to own an object in the future, one establishes a psychological ownership of the object^75,76^. This is the first study that manipulates the psychological ownership of a placebo, no prior research has used this type of manipulation before. Although the results of this study demonstrated that the effect of psychologically possessing a placebo analgesic was not significantly different from that of no possession, it is too early to conclude that psychological possession is not important for placebo analgesia. In the current study, participants were healthy college students who came to do the cold pressor test and did not anticipate the pain to persist past the experiment. However, in the context of persistent or chronic pain, having a prescription for an analgesic agent (i.e., psychological possession) may become important and powerful as it holds promise for relieve future pain.

In conclusion, having tangible medication available (without use) could be sufficient to create a difference in pain perception and tolerance; and the physical ownership of analgesic might have impact on the anticipation of future pain. The current study constitutes an initial attempt to examine the influence of temporal suggestion implicated by the form of possession on placebo effect. It provides insight into the time course of the possession-induced analgesic effect. A new wave of studies integrating the possession effect and placebo effect could offer an innovative frontier for theory, research, and practice. Future direction of this line of research could examine the longevity of the possession effect and its impact on other persistent health-related symptoms, for example, smoking, depression, stress, and anxiety.

## Supplementary Information

Supplementary Information.
