# Supplementary material for: Temporal expectancy induced by the mere possession of a placebo analgesic affects placebo analgesia: preliminary findings from a randomized controlled trial
Source: Sci Rep. 2022 Jan 26;12:1395. doi: 10.1038/s41598-022-05537-9 (PMC8792021; doi:10.1038/s41598-022-05537-9)
Supplement: Supplementary file 1 — Supplementary Information. [file 41598_2022_5537_MOESM1_ESM.docx]

**Temporal expectancy induced by the mere possession of a placebo analgesic affects placebo analgesia: Preliminary findings from a randomized controlled trial**

**Supplementary Materials**

**Scales used in online survey to assess personality variables**

During the online registration, participants who were eligible to participate in the study were directed to complete a questionnaire measuring their personality variables. All scales were in Chinese. We adopted the well-established translated version if it was available, if not, we translated it by ourselves with procedures of back-translation to ensure validity. These included Fear of Pain Questionnaire – III, Desirability for Control Scale, Short Suggestibility Scale, State-Trait Anxiety Inventory-Trait Scale (STAI-Y2), Pain Catastrophizing Scale. Rosenberg Self-esteem Scale, Private Self-consciousness Scale, Somatosensory Amplification Scale, Motivational Persistence Scale. The presentation order of the scales and the scale items were randomized. Justifications for including these personality variables are presented below:

***Fear of pain***

Prior research (Lyby et al., 2010) found that fear of pain was positively correlated to stress during/anticipating pain; and negatively correlated to placebo analgesia. Also, increased fear of pain reduced subjective and electrophysiological placebo analgesic responses (Lyby et al., 2011). It was also found that when fear was induced to participants, especially those scored high on measures of pain, placebo analgesia was abolished (Lyby et al., 2012). Furthermore, dispositional fear of pain predicted nocebo hyperalgesia (Aslaksen & Lyby, 2015). As such, in our study, fear of pain was measured.

***Desirability for control***

Participants high in desirability of control and high sense of control experienced greater placebo analgesia (Feldner & Hekmat, 2001; Geers et al., 2010). Those who chronically desired to control experienced less pain in experimental pain task when they have the freedom to decide their placebo analgesic treatments (Geers et al., 2013). We expect that participants high in desire for control will experience placebo analgesia when they physically possessed and felt having control over the placebo analgesic essential oil.

***Suggestibility***

Participants high in suggestibility were more likely to prone to illusion or accept suggestions (De Pascalis et al., 2002). We expect participants will be more likely to enjoy placebo analgesia when they physically possessed a placebo analgesic essential oil and accepted the suggestion that it is an effective analgesic that can offer immediate pain relief.

***Trait anxiety***

Past research (Tang & Gibson, 2005) reported that participants having high trait anxiety were more likely to exacerbate the perceived pain stimulations than participants having low trait anxiety. State trait anxiety score was also significantly correlated with nocebo responses (Colloca et al., 2010). High trait anxiety also altered placebo analgesia (Grös et al., 2010). We thus measured participants’ trait anxiety.

***Catastrophic thinking tendency***

Catastrophic thoughts affected pain sensation (Sullivan et al., 1995; Sullivan & Neish, 2001). Participants engaged in catastrophic thoughts were more attentive to pain signals (Sullivan et al., 2001) and exaggerate pain seriousness, subsequently leading to greater physical pain (Sullivan & Neish, 2001). In addition, pain catastrophizing thought tendency was positively correlated to NRS and SF-MPQ pain ratings (France et al., 2002), and predicted poorer response to topical analgesics (Mankovsky et al., 2012). We expect that participants low in catastrophic thoughts would enjoy placebo analgesia.

***Self-esteem***

Prior research (De Angelis et al., 2012; Jones, 1973; Shrauger, 1975) reported that participants low in self-esteem used self-enhancing experiences to boost their self-esteem. We expect the physical possession of a placebo analgesic essential oil provided an experience to boost up participants’ self-efficacy during CPT.

***Private self-consciousness***

In social psychology literature, mere ownership/possession effect refers to the cognitive tendency to associate the self and the positive attributes of the owned object. Beggan (1992) explained that this cognitive tendency was an attempt to enhance private (vs. public) self-image. Morewedge and Giblin (2015) proposed that the ownership effect was moderated by private self-consciousness. We expect that the higher the participants’ private self-consciousness (i.e., more involvement in self-reflective thought), the more susceptible they will be to the mere possession effect, i.e., trying to associate the self more strongly to the owned analgesic essential oil and thus increase pain resilience.

***Somatosensory amplification***

Somatosensory amplification was closely connected to clinical pain (Gregory et al., 2005) and experimentally induced pain (Ferentzi et al., 2017; Lee et al., 2010). Also, participants high (vs. low) in somatosensory amplification and trait anxiety showed greater pain-perception (Cioffi et al., 2016). We expect participants high in somatosensory amplifications will show lower pain analgesia.

***Motivational persistence***

Motivational persistence is the tendency to continue engaging in a task despite difficulties (Constantin et al., 2012). Participants having high persistence at a difficult motor control task were more likely to succeed at other self-control task (Brandon et al., 2003; Kieras et al., 2005; Schmeichel & Zell, 2007). We expect participants high in motivational persistence will perform better in self-control tasks such as a CPT.

**Materials**

***Fear of Pain Questionnaire – III.***

The 30-item Fear of pain Questionnaire-III (McNeil & Rainwater, 1998, translated by Yeung et al., 2019) was adopted to measure participants’ fear of pain. Participants rated how fearful they were when experiencing the pain described (e.g. “Having a muscle cramp”) on a 5-point scale (1 = “Not at all”, 5 = “Extreme”). Mean score was computed for each participant (Cronbach’s α = .93).

***Desirability for Control Scale.***

Participants’ desire for control was measured by the Desirability for Control Scale (Burger & Cooper, 1979). The scale originally consists twenty items but three items were excluded as they were not appropriate to our sample. Among the seventeen items, five items were negatively worded (e.g. “I wish I could push many of life’s daily decisions off on someone else.”). Participants rated whether the description in the statements apply to them on a 7-point scale (1 = “The statement does not apply to me at all”, 7 = “The statement always applies to me”). Mean score was obtained after the five negative statements were reversely coded (Cronbach’s α = .79).

***Short Suggestibility Scale.***

The 21-item Short Suggestibility Scale from the Multidimensional Iowa Suggestibility Scale (Kotov et al., 2004) was used to measure participants’ suggestibility. On a 7-point scale (1 = “strongly disagree”, 7 = “strongly agree”), participants rated whether the statements (e.g. “Imagining a refreshing drink can make me thirsty.”) apply to them. All twenty-one items are positively worded and mean score was computed for each participant (Cronbach’s α = .92).

***State-Trait Anxiety Inventory-Trait Scale (STAI-Y2).***

To measure participants’ trait anxiety level, the Trait-Anxiety scale of the State-Trait Anxiety Inventory (Spielberger et al., 1983, translated by Yeung et al., 2019) was used. Nine items indicated absence of anxiety (e.g. “I feel secure.”), and eleven items indicated presence of anxiety (e.g. “I feel like a failure.”). Participants rated the degree of which the statements could apply to them in general on a 4-point scale (1 = “Almost never”, 4 = “Almost always”). Scores in the anxiety-absent items were reversed then added to the scores of anxiety-present items. Mean score was computed for each participant (Cronbach’s α = .88).

***Pain Catastrophizing Scale.***

The Pain Catastrophizing Scale (Sullivan et al., 1995) was used to assess participants’ catastrophizing thinking towards pain. Participants rated the extent to which they experienced each of the 13 situations (e.g. “I anxiously want the pain to go away”) on a 5-point scale (1 = “Not at all”, 5 = “All the time”). Mean score was obtained for each participant (Cronbach’s α = .93).

***Rosenberg Self-esteem Scale.***

The 10-item Rosenberg Self-esteem Scale (Rosenberg, 1965, translated by Leung & Wong, 2008) was adopted to measure participants’ level of self-esteem. Participants rated their agreement to each statement on a 4-point scale (1 = “Strongly agree”, 4 = “Strongly disagree”). Five statements are positively worded (e.g. “I take a positive attitude toward myself.”), and five statements are negatively worded (e.g. “I certainly feel useless at times.”). Mean score was obtained by averaging all ten items after the negatively worded items were reversely coded (Cronbach’s α = .78).

***Private Self-consciousness Scale.***

The 10-item private Self-consciousness scale from the Self-Consciousness Inventory (Fenigstein et al., 1975) was used to measure respondents’ tendency of focusing on aspects of self. Participants rated how well the statements described them on a 5-point scale (1 = “Extremely uncharacteristic”, 5 = “Extremely characteristic”). Two items are negatively worded (e.g. “I never scrutinize myself.”), and eight items are positively worded (e.g. “I reflect about myself a lot.”). Mean score was computed by averaging all ten items after the negatively worded items were reversely coded (Cronbach’s α = .77).

***Somatosensory Amplification Scale.***

The 10-item Somatosensory Amplification Scale (Barsky et al., 1990) was adopted to measure participants’ tendency to intensify somatic sensations. Participants rated the extent to which each of the statements (e.g. “I hate to be too hot or too cold.”) correctly described them in general on a 5-point scale (1 = “Not at all true”, 5 = “Extremely true”). Mean score was obtained by averaging the scores of ten items, with higher scores mean higher tendency to intensify sensations (Cronbach’s α = .77).

***Motivational Persistence Scale.***

The 16-item Motivational Persistence Scale developed by Constantin et al. (2012) was used to measure participants’ level of persistence. Participants indicated the degree to which each item (e.g. “The more difficult a task is, the more determined I am to finish it”) describes them on a 5-point scale, (1 = “A very low degree”, 5 = “A very high degree”). Mean score was obtained for each participant (Cronbach’s α = .86).

**Results and Discussion**

A one-way ANOVA was conducted with possession condition (physical vs. psychological vs. no) as the independent variable and each of the personality variables as the dependent variable. A marginally significant main effect of possession condition was found in participants’ desire for control score, and a significant main effect of possession condition was found in their suggestibility and self-esteem scores, respectively. Turkey HSD revealed that the participants in the physical-possession condition showed having slightly lower desire for control than participants in the psychological-possession, *p*=.06, but not differed from those in the no-possession condition, *p*=.94. There was no significant difference between psychological-possession condition and no-possession condition in desire for control, *p*=.17. Moreover, Tukey HSD showed that participants in the physical-possession condition were slightly higher in their suggestibility score than those in the no-possession condition, *p*=.06, but not differed from those in the psychological-possession, *p*=.12. Participants in psychological-possession condition and no-possession did not differ statistically in suggestibility, *p*=.96. Finally, participants in the physical-possession condition have slightly lower self-esteem than participants in the psychological-possession condition, *p*=.09, and that of no-possession condition, *p* =.09. And there was no significant difference between psychological and no possession conditions in self-esteem, *p*=1.00.

In general, our data showed that the main effect of possession condition was not significant for most of the tested personality variables, all *p*s = ns. This also indicates that participants did not have any significant pre-existing personality difference before experimental possession manipulation. The means, standard deviations and *F*-statistics of the corresponding variables are presented in Table A below.

*Table A*. Means, standard deviations (in parentheses), and *F*-statistics of personality variables across the three possession conditions.

| Measure | Scale | Physical-Possession  (*n*=30) | Psychological-Possession  (*n*=29) | No-Possession  (*n*=29) | *F*-statistics ^a^ | *Effect size (*$\eta_{p}^{2})$ | *Post-Hoc* |
| --- | --- | --- | --- | --- | --- | --- | --- |
| Fear of Pain (FOPQ-III) | 1-5 | 3.30 (.5.8) | 3.41 (.67) | 3.25 (.56) | .56 | .01 |  |
| Desire for Control (DCS) | 1-7 | 4.33 (.59) | 4.67 (.63) | 4.38 (.53) | 3.00 ^†^ | .07 | Phy<Psy (*p*=.06)  Phy=No  Psy=No |
| Suggestibility (SSS) | 1-7 | 4.44 (.73) | 4.00 (.81) | 3.94 (.95) | 3.14* | .07 | Phy>No (*p*=.06)  Phy=Psy  Psy=No |
| Trait Anxiety (STAI-Y2) | 1-4 | 2.43 (.39) | 2.67 (.46) | 2.28 (.36) | 1.00 | .37 |  |
| Pain Catastrophizing (PCS) | 1-5 | 2.87 (.71) | 2.86 (.76) | 2.82 (.76) | .03 | .001 |  |
| Rosenberg Self-esteem | 1-4 | 2.53 (.39) | 2.73(.38) | 2.72 (.28) | 3.06* | .07 | Phy< Psy (*p*=.09)  Psy < No (*p*=.09)  Psy=No |
| Private self-consciousness | 1-5 | 3.41 (.52) | 3.54 (.64) | 3.48 (.50) | .43 | .01 |  |
| Somatosensory Amplification | 1-5 | 3.04 (.69) | 2.90 (.59) | 2.94 (.62) | .37 | .009 |  |
| Motivational Persistence | 1-5 | 3.22 (.55) | 3.27 (.43) | 3.25 (.46) | .08 | .002 |  |

*Note*. Phy=physical possession, Psy=psychological possession, No=no possession;

^a^ For univariate *F*-tests, degrees of freedom are (2,85).

**p* < .05. ^†^ *p* = .055

**Evaluation of the presented placebo analgesic essential oil**

Participants were instructed to complete a purported marketing survey in which they evaluated the presented (placebo) analgesic essential oil in terms of their purchase intention, general impression and attitude of essential oil product, likeness of the packaging design, and estimated price for the essential oil product if it was available in Hong Kong market. All these aimed to mask the real purpose of the experiment and make it more resemble a marketing survey. The means, standard deviations, and *F*-statistics of the analgesic essential oil related variables are presented in Table B.

**Results and discussion**

ANOVAs were conducted with possession condition as the independent variables and each of the product related variables as the dependent variable. Results found no significant main effect of possession condition in all variables, all *p*s=ns. Participants shared similar evaluations of the presented placebo analgesic during the purported marketing survey (see Table B below).

*Table B*. The means, standard deviations, and *F*-statistics of the analgesic cream related variables (from purported marketing survey).

| Measure | Scale | Physical-Possession  (*n*=30) | Psychological-Possession  (*n*=29) | No-Possession  (*n*=29) | *F*-statistics ^a^ | *Effect size (*$\eta_{p}^{2})$ |
| --- | --- | --- | --- | --- | --- | --- |
| Use-intention | 1-7 | 4.10 (1.32) | 4.10 (1.21) | 4.31 (1.34) | .25 | .006 |
| Purchase-intention | 1-7 | 4.63 (1.35) | 4.86(1.25) | 4.62 (1.21) | .33 | .008 |
| Package design | 1-7 | 4.53 (1.04) | 4.72 (1.28) | 4.38 (1.35) | .57 | .013 |
| General impression | 1-7 | 4.97 (.89) | 5.17 (1.04) | 5.03 (1.02) | .33 | .008 |
| Estimated price | Any $ | 142.06 (81.37) | 144.62 (119.73) | 170.69 (134.50) | .57 | .013 |
| Attitude (oil) | 1-10 | 4.30 (1.24) | 4.55 (1.12) | 4.28 (1.44) | .42 | .01 |
| Attitude (analgesic) | 1-10 | 4.20 (1.47) | 4.14 (1.06) | 3.93 (.88) | .43 | .01 |

*Note*. Values outside parentheses are means, inside are standard deviations.

^a^ For univariate *F*-tests, degrees of freedom are (2, 85).

**Correlational data**

Since participants did not show significant difference in most of the personality variables and product evaluation variables, we conducted correlational analyses using the full data set (*N*=88) to examine whether our personality variables and product evaluation variables were correlated with our physical pain outcomes (i.e., pain threshold and pain tolerance). Table C displays the correlation coefficients. We found that all variables were not significantly correlated with the physical pain outcomes, except for fear of pain. Our data showed that fear of pain was negatively correlated to pain threshold and pain tolerance, meaning that the more the participants felt fear of pain, the faster they felt the pain sensation on their skin and the quicker they withdrew their hand from the cold water. This echo the past research findings that fear of pain was negatively related to analgesia (Lyby et al., 2010).

*Table C*. Correlations between personality variables and product evaluation variables with physical pain outcomes.

|  | Pain threshold | Pain tolerance |
| --- | --- | --- |
| Fear of Pain (FOPQ-III) | -.21* | -.28** |
| Desire for Control (DCS) | -.07 | .04 |
| Suggestibility (SSS) | .06 | .15 |
| Trait Anxiety (STAI-Y2) | -.04 | .05 |
| Pain Catastrophizing (PCS) | -.10 | -.12 |
| Rosenberg Self-esteem | -.06 | -.17 |
| Private self-consciousness | .05 | .006 |
| Somatosensory Amplification | -.01 | -.15 |
| Motivational Persistence | -.05 | .09 |
| Use-intention | -.01 | -0.14 |
| Purchase-intention | -.09 | -.03 |
| Package design | .07 | -.13 |
| General impression | -.11 | -.07 |
| Estimated price | -.09 | .07 |
| Attitude (oil) | -.02 | .03 |
| Attitude (analgesic) | .10 | .07 |

** Correlation is significant, p<.001 (2-tailed).

* Correlation is significant, p<.05 (2-tailed).

**References**

Aslaksen, P. M., & Lyby, P. S. (2015). Fear of pain potentiates nocebo hyperalgesia. *Journal of Pain Research*, *8*, 703–710. https://doi.org/10.2147/JPR.S91923

Barsky, A. J., Wyshak, G., & Klerman, G. L. (1990). The somatosensory amplification scale and its relationship to hypochondriasis. *Journal of Psychiatric Research*, *24*(4), 323–334. https://doi.org/10.1016/0022-3956(90)90004-a

Beggan, J. K. (1992). On the social nature of nonsocial perception: The mere ownership effect. *Journal of Personality and Social Psychology*, *62*(2), 229–237. https://doi.org/10.1037/0022-3514.62.2.229

Brandon, T., Herzog, T., Juliano, L., Irvin, J., Lazev, A., & Simmons, V. (2003). Pretreatment task persistence predicts smoking cessation outcome. Journal of Abnormal Psychology, 112, 448-456. *Journal of Abnormal Psychology*, *112*, 448–456. https://doi.org/10.1037/0021-843X.112.3.448

Burger, J. M., & Cooper, H. M. (1979). The desirability of control. *Motivation and Emotion*, *3*, 381–393.

Cioffi, I., Michelotti, A., Perrotta, S., Chiodini, P., & Ohrbach, R. (2016). Effect of somatosensory amplification and trait anxiety on experimentally induced orthodontic pain. *European Journal of Oral Sciences*, *124*(2), 127–134. https://doi.org/10.1111/eos.12258

Colloca, L., Petrovic, P., Wager, T. D., Ingvar, M., & Benedetti, F. (2010). How the number of learning trials affects placebo and nocebo responses. *Pain*, *151*(2), 430–439. https://doi.org/10.1016/j.pain.2010.08.007

Constantin, T., Holman, A., Ana Maria, H., & Hojbotǎ, A. M. (2012). Development and validation of a motivational persistence scale. *Psihologija*, *45*(2), 99–120. https://doi.org/10.2298/PSI1202099C

De Angelis, M., Bonezzi, A., Peluso, A. M., Rucker, D. D., & Costabile, M. (2012). On Braggarts and Gossips: A Self-Enhancement Account of Word-of-Mouth Generation and Transmission. *Journal of Marketing Research*, *49*(4), 551–563. https://doi.org/10.1509/jmr.11.0136

De Pascalis, V., Chiaradia, C., & Carotenuto, E. (2002). The contribution of suggestibility and expectation to placebo analgesia phenomenon in an experimental setting. *Pain*, *96*(3), 393–402. https://doi.org/10.1016/S0304-3959(01)00485-7

Feldner, M. T., & Hekmat, H. (2001). Perceived control over anxiety-related events as a predictor of pain behaviors in a cold pressor task. *Journal of Behavior Therapy and Experimental Psychiatry*, *32*(4), 191–202. https://doi.org/10.1016/S0005-7916(01)00034-9

Fenigstein, A., Scheier, M. F., & Buss, A. H. (1975). Public and private self-consciousness: Assessment and theory. *Journal of Consulting and Clinical Psychology*, *43*(4), 522–527. https://doi.org/10.1037/h0076760

Ferentzi, E., Köteles, F., Csala, B., Drew, R., Tihanyi, B. T., Pulay-Kottlár, G., & Doering, B. K. (2017). What makes sense in our body? Personality and sensory correlates of body awareness and somatosensory amplification. *Personality and Individual Differences*, *104*, 75–81. https://doi.org/10.1016/j.paid.2016.07.034

France, C. R., France, J. L., al’Absi, M., Ring, C., & McIntyre, D. (2002). Catastrophizing is related to pain ratings, but not nociceptive flexion reflex threshold. *Pain*, *99*(3), 459–463. https://doi.org/10.1016/S0304-3959(02)00235-X

Geers, A. L., Rose, J. P., Fowler, S. L., Rasinski, H. M., Brown, J. A., & Helfer, S. G. (2013). Why does choice enhance treatment effectiveness? Using placebo treatments to demonstrate the role of personal control. *Journal of Personality and Social Psychology*, *105*(4), 549–566. https://doi.org/10.1037/a0034005

Geers, A. L., Wellman, J. A., Fowler, S. L., Helfer, S. G., & France, C. R. (2010). Dispositional optimism predicts placebo analgesia. *Journal of Pain*, *11*(11), 1165–1171. https://doi.org/10.1016/j.jpain.2010.02.014

Gregory, R. J., Manring, J., & Wade, M. J. (2005). Personality traits related to chronic pain location. *Annals of Clinical Psychiatry*, *17*(2), 59–64. https://doi.org/10.1080/10401230590932317

Grös, D. F., Simms, L. J., & Antony, M. M. (2010). Psychometric Properties of the State-Trait Inventory for Cognitive and Somatic Anxiety (STICSA) in Friendship Dyads. *Behavior Therapy*, *41*(3), 277–284. https://doi.org/10.1016/j.beth.2009.07.001

Jones, S. C. (1973). Self- and interpersonal evaluations: Esteem theories versus consistency theories. *Psychological Bulletin*, *79*(3), 185–199. https://doi.org/10.1037/h0033957

Kieras, J. E., Tobin, R. M., Graziano, W. G., & Rothbart, M. K. (2005). You can’t always get what you want effortful control and children’s responses to undesirable gifts. *Psychological Science*, *16*(5), 391–396. https://doi.org/10.1111/j.0956-7976.2005.01546.x

Kotov, R. I., Bellman, S. B., & Watson, D. B. (2004). *Multidimensional Iowa suggestibility scale (MISS)*. http://medicine.stonybrookmedicine.edu/system/files/MISS_FINAL_BLANK_0.pdf

Lee, J. E., Watson, D., & Frey Law, L. A. (2010). Lower-Order Pain-Related Constructs Are More Predictive of Cold Pressor Pain Ratings than Higher-Order Personality Traits. *Journal of Pain*, *11*(7), 681–691. https://doi.org/10.1016/j.jpain.2009.10.013

Leung, S. O., & Wong, P. M. (2008). Validity and reliability of Chinese Rosenberg Self-Esteem Scale. *New Horizons in Education*, *56*, 62–69.

Lyby, P. S., Aslaksen, P. M., & Flaten, M. A. (2010). Is fear of pain related to placebo analgesia? *Journal of Psychosomatic Research*, *68*(4), 369–377. https://doi.org/10.1016/j.jpsychores.2009.10.009

Lyby, P. S., Aslaksen, P. M., & Flaten, M. A. (2011). Variability in placebo analgesia and the role of fear of pain - An ERP study. *Pain*, *152*(10), 2405–2412. https://doi.org/10.1016/j.pain.2011.07.010

Lyby, P. S., Forsberg, J. T., Åsli, O., & Flaten, M. A. (2012). Induced fear reduces the effectiveness of a placebo intervention on pain. *Pain*, *153*(5), 1114–1121. https://doi.org/10.1016/j.pain.2012.02.042

Mankovsky, T., Lynch, M., Clark, A., Sawynok, J., & Sullivan, M. J. L. (2012). Pain catastrophizing predicts poor response to topical analgesics in patients with neuropathic pain. *Pain Research & Management*, *17*(1), 10–14. https://doi.org/10.1155/2012/970423

McNeil, D. W., & Rainwater, A. J. 3rd. (1998). Development of the Fear of Pain Questionnaire--III. *Journal of Behavioral Medicine*, *21*(4), 389–410. https://doi.org/10.1023/a:1018782831217

Morewedge, C. K., & Giblin, C. E. (2015). Explanations of the endowment effect: an integrative review. *Trends in Cognitive Sciences*, *19*(6), 339–348. https://doi.org/10.1016/j.tics.2015.04.004

Rosenberg, M. (1965). Rosenberg self-esteem scale (RSE). *Acceptance and Commitment Therapy. Measures Package*, *61*(52), 18.

Schmeichel, B. J., & Zell, A. (2007). Trait Self‐Control Predicts Performance on Behavioral Tests of Self‐Control. *Journal of Personality*, *75*(4), 743–756. https://doi.org/10.1111/j.1467-6494.2007.00455.x

Shrauger, J. S. (1975). Responses to evaluation as a function of initial self-perceptions. *Psychological Bulletin*, *82*(4), 581–596. https://doi.org/10.1037/h0076791

Spielberger, C., Gorsuch, R., Lushene, R., Vagg, P. R., & Jacobs, G. (1983). Manual for the State-Trait Anxiety Inventory (Form Y1 – Y2). In *Palo Alto, CA: Consulting Psychologists Press; Vol. IV*.

Sullivan, M. J. L., Bishop, S. R., & Pivik, J. (1995). The Pain Catastrophizing Scale: Development and Validation. *Psychological Assessment*, *7*(4), 524–532. https://doi.org/10.1037/1040-3590.7.4.524

Sullivan, M. J. L., & Neish, N. (2001). Catastrophic thinking and the experience of pain during dental procedures. *Journal (Indiana Dental Association)*, *79*(4), 16–19.

Sullivan, M. J. L., Thorn, B., Haythornthwaite, J. A., Keefe, F., Martin, M., Bradley, L. A., & Lefebvre, J. C. (2001). Theoretical perspectives on the relation between catastrophizing and pain. *The Clinical Journal of Pain*, *17*(1), 52–64. https://doi.org/10.1097/00002508-200103000-00008

Tang, J., & Gibson, S. J. (2005). A psychophysical evaluation of the relationship between trait anxiety, pain perception, and induced state anxiety. *Journal of Pain*, *6*(9), 612–619. https://doi.org/10.1016/j.jpain.2005.03.009

Yeung, V. W.L, Geers, A. L., & Kam, S. M. C. (2019). Merely Possessing a Placebo Analgesic Reduced Pain Intensity: Preliminary Findings from a Randomized Design. *Current Psychology*, *38*(1), 194–203. https://doi.org/10.1007/s12144-017-9601-0
